# Constitutive activation and overexpression of NF-κB/c-Rel in conjunction with p50 contribute to aggressive tongue tumorigenesis

**DOI:** 10.18632/oncotarget.26041

**Published:** 2018-08-31

**Authors:** Shilpi Gupta, Prabhat Kumar, Harsimrut Kaur, Nishi Sharma, Sunita Gupta, Daman Saluja, Alok C. Bharti, Bhudev Das

**Affiliations:** ^1^ Stem Cell and Cancer Research Lab, Amity Institute of Molecular Medicine and Stem Cell Research, Amity University Uttar Pradesh, Sector-125, Noida-201313, India; ^2^ Dr. B. R. Ambedkar Center for Biomedical Research, University of Delhi, Delhi-110007, India; ^3^ Department of Otorhinolaryngology, Post Graduate Institute of Medical Education and Research, Dr. Ram Manohar Lohia Hospital, Delhi-110010, India; ^4^ Department of Oral Medicine and Radiology, Maulana Azad Institute of Dental Sciences, Delhi-110010, India; ^5^ Molecular Oncology Laboratory, Department of Zoology, University of Delhi, Delhi-110007, India

**Keywords:** tongue squamous cell carcinoma, NF-κB/c-Rel, Fra-2, HPV, prognosis

## Abstract

Tongue squamous cell carcinoma (TSCC) is a most aggressive head and neck cancer often associated with a poor survival rate. Yet, it always shows better prognosis in presence of HPV16 infection. NF-κB plays a pivotal role in carcinogenesis and chemo-radio resistance of cancer but its role in tongue cancer is not yet explored. In this study, a total of hundred tongue tissue biopsies comprising precancer, cancer and adjacent normal controls including two tongue cancer cell lines (HPV^+/−ve^) were employed to examine expression and transactivation of NF-κB proteins, their silencing by siRNA and invasion assays to understand their contributions in tongue carcinogenesis. An exclusive prevalence (28%) of HR-HPV type 16 was observed mainly in well differentiated tumors (78.5%). Increased DNA binding activity and differential expression of NF-κB proteins was observed with p50 and c-Rel being the two major DNA binding partners forming the functional NF-κB complex that increased as a function of severity of lesions in both HPV^+/−ve^ tumors but selective participation of p65 in HPV16^+ve^ TSCCs induced well differentiation of tumors resulting in better prognosis. siRNA treatment against c-Rel or Fra-2 led to upregulation of p27 but strong inhibition of c-Rel, c-Jun, c-myc, HPVE6/E7 and Fra-2 which is exclusively overexpressed in HPV^−ve^ aggressive tumors. In conclusion, selective participation of c-Rel with p50 that in cross-talk with AP-1/Fra-2 induced poor differentiation and aggressive tumorigenesis mainly in HPV^−ve^ smokers while HPV infection induced expression of p65 and p27 leading to well differentiation and better prognosis preferably in non-smoking TSCC patients.

## INTRODUCTION

Tongue squamous cell carcinoma (TSCC) is one of the most common but a lethal head and neck malignancy [1]. It is a highly aggressive cancer with poor prognosis mostly prevalent in younger subjects [2–6]. Inspite of enormous advances in diagnosis and treatment, there is not much improvement in survival rate of TSCC patients. Therefore, understanding pathogenesis of tongue cancer is important to identify potential diagnostic and therapeutic target(s) for better management of the disease. Numerous clinico-epidemiological and molecular studies have established that the high incidence of TSCC is mainly associated with tobacco and alcohol consumption [7, 8] but infection with high-risk human papillomaviruses (HR-HPVs) is also recognized as an independent risk-factor [9]. Interestingly, head and neck squamous cell carcinomas (HNSCCs) including TSCCs always show a good prognosis and better survival in presence of HPV infection [3, 4, 6, 10–13]. However, it is not well understood how viral oncogenes regulate cellular and molecular pathogenic events that lead to better prognosis when treated.

The pro-inflammatory transcription factor NF-κB plays a pivotal role in regulating expression of genes and pathways that play a key role in initiation and progression of various cancers including tongue cancer. NF-κB has family of five major proteins (NF-κB1/p50, NF-κB2/p52, RelA/p65, c-Rel and RelB) that form a functional homo/heterodimeric complex and the most abundant and active form is a heterodimer composed of p50 and p65 proteins [14]. The Rel family of proteins RelA, RelB and c-Rel possess a c-terminal domain responsible for transcriptional activation. Inflammatory response often activates NF-κB which gets translocated on to the nucleus and up or down several downstream gene expression. Numerous studies have reported differential expression and higher DNA binding activity of NF-κB in different stages of various cancers [12, 15–19] including oral cancer [12] and it also leads to chemo-radiotherapy resistance, and poor prognosis [19]. An elevated level of c-Rel expression has also been shown to be associated with aggressive tumor phenotype [12, 20–22]. Either homo or hetero-dimerization of c-Rel with p50 has been reported to be associated with aggressive B- and T-cell lymphomas [23]. While it leads to the induction of apoptosis yet ectopic expression of c-Rel promoted therapy resistance [24]. In contrast, oral cancer often showed preferential p50/p50 homo-dimerization but HR-HPV type 16 infection promoted selective participation of p65 leading to well differentiation and better prognosis of tumor [12]. These observations strongly suggest that NF-κB proteins represent a class of potential marker(s) for the progression and/or prognosis of oral tongue cancer. In this study, we report first time a differential functional regulatory role of NF-κB/c-Rel/p65 and their target genes in presence or absence of HPV infection in a spectrum of tongue cancer tissues and cell lines and their role in tumor aggressiveness and drug resistance. We also show that c-Rel in crosstalk with AP-1/Fra-2, can induce aggressive tumor phenotype and poor prognosis in absence of HPV mostly in patients with tobacco habits while HPV16 infection induced well differentiation and better prognosis mostly in non-smoking TSCC patients.

## RESULTS

To molecularly dissect the role of NF-κB in TSCC, prospectively collected one hundred fresh tongue tissue biopsies comprising precancer (*n* = 20), cancer (*n* = 50) and adjacent normal controls (*n* = 30) and a HPV^+ve^ (UPCI:SCC090) and a HPV^−ve^ (AW13516) tongue cancer cell lines were analyzed. Of twenty precancer cases, 11 (55%) were either leukoplakia or erythroplakia and 9 (45%) were dysplasia (Table 1A). DNA isolated from tissue specimens and cell lines were subjected to HPV detection and HPV genotyping while protein and RNA extracted were used for analysis of expression and DNA binding activity of NF-κB family proteins. The clinico-epidemiological and demographic details along with HPV status of all patients are presented in Table 1A. The results demonstrate that the majority of cancer patients were smokers (84%; 42/50) and males (40/50; 80%) with a mean age of 40.48 ± 12.46 years but 60% patients were below 35 years of age with advanced tumor grades (T3/T4; 60%) and stages (III/IV; 72%), and node negative. Out of 50 tongue cancer cases, 15 (30%) were well differentiated squamous cell carcinomas (WDSCCs), 8 (16%) were moderately differentiated (MDSCCs) while 27 (54%) were poorly differentiated squamous cell carcinomas (PDSCCs).

**Table 1A:** Clinico-pathological and demographic characteristics and their correlation with HPV16 infection in tongue cancer patients

Characteristics		No. of cases (%)	Total HPV positive	HPV negative	*p*-values
Adjacent normal controls		30	nil	-	-
**Precancer (*n* = 20)**	**36.7 ± 5.99**	**Mean age ± SD**	**nil**	-	
Precancer (*n* = 20)	Leukoplakia/Erthroplakia	11 (55%)	nil	-	- -
	Dysplasia	9 (45%)	nil	-	
**Cancer (*n* = 50)**	**40.48 ± 12.46**	**Mean age ± SD**	**14 (28%)**	**36 (72%)**	
**Age ranges**	<35	30 (60%)	10 (71.4%)	20 (55.6%)	0.3 (ns)
	>35	20 (40%)	4 (28.6%)	16 (44.4%)	
**Gender**	Male	40 (80%)	6 (42.9%)	34 (94.4%)	**0.0002**
	**Female**	**10 (20%)**	**8 (57.2%)**	**2 (5.6%)**	
**Religion**	Hindu	40 (80%)	13 (92.9%)	27 (75%)	0.2 (ns)
	Muslim	10 (20%)	1 (7.1%)	9 (25%)	
**Addiction habits**	**Tobacco smokers**	**42 (84%)**	**8 (57.1%)**	**34 (94.4%)**	**0.003**
	Non-smokers	8 (16%)	6 (42.9%)	2 (5.6%)	
**Tumor site**	**Base of tongue**	22 (44%)	**12 (85.7%)**	10 (27.8%)	**0.0003**
	Mobile tongue & other sites of tongue	28 (56%)	2 (14.3%)	26 (72.2%)	
**Differentiation status**	**WDSCC**	15 (30%)	**11 (78.6%)**	4 (11.2%)	**0.0001**
	MDSCC	8 (16%)	2 (14.3%)	6 (16.7%)	
	PDSCC	27 (54%)	1 (7.1%)	26 (72.2%)	
**Tumor status**	**T1–T2**	20 (40%)	**11 (78.6%)**	9 (25%)	**0.0009**
	T3–T4	30 (60%)	3 (21.4%)	27 (75%)	
**Node status**	**N0–N1**	39 (78%)	**13 (92.9%)**	26 (72.2%)	0.2 (ns)
	N2–N3	11 (22%)	1 (7.1%)	10 (27.8%)	
**Clinical staging**	Stage I–II	14 (28%)	10 (71.4%)	4 (11.1%)	**0.0001**
	Stage III–IV	36 (72%)	4 (28.6%)	32 (88.9%)	

n: number of patients. T: primary tumor. N: regional lymph node. All cases comprised adjacent normal controls, precancer, HPV^−ve^ and HPV16^+ve^ cancer patients. *p-values* obtained by probability Fisher's (exact) test using Graph Pad Prism 6.0. *p-values* ≤ 0.05 are considered as statistically significant. ns = not significant.

### Exclusive prevalence of HPV 16 infection mainly in non-smoking patients

DNA extracted from tissue biopsy specimens comprising precancer, cancer and adjacent normal controls including two cell lines were tested for the presence of HPV infection, first by consensus primers, then by HPV type-specific PCR followed by reverse line blot assay (RLB) which revealed 14 TSCCs (28%) exclusively positive for HR-HPV type 16 and rest all cases were found to be negative for HPV (Table 1A and Supplementary Figure 1A–1C). The majority of HPV^+ve^ TSCCs (57.2%; 8/14, *p* = 0.0002) were females who showed well differentiated tumors (see Table 1A) of early grades (stage I-II). Interestingly, a significantly higher prevalence of HPV infection was observed in non-tobacco smoking (75%, 6/8; *p* ≤ 0.01) TSCC patients who were mainly (75%; 6/8) women while majority were smokers and negative for HPV infection (94.4%; *p* = 0.003). In contrast, majority of TSCC patients were males (80%) and all were smokers but only 15% (6/40) of them were positive for HPV infection (Supplementary Figure 1D).

### Higher DNA binding activity and transactivation of NF-κB contributed by preferential heterodimerization of p50/c-Rel during tongue carcinogenesis

**Figure 1:**
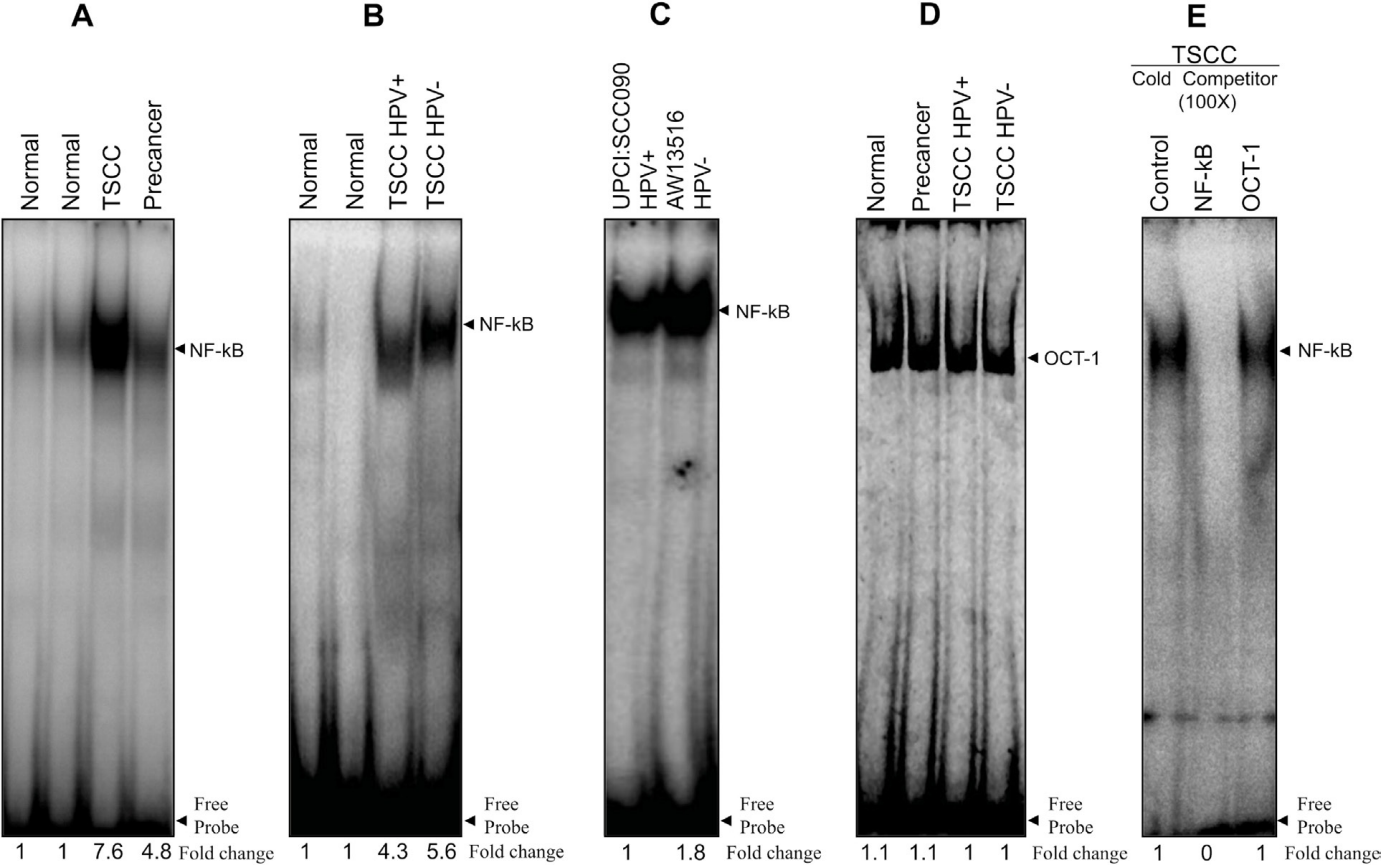
(**A**–**E**) Higher NF-κB DNA binding activity in HPV^+ve^ and HPV^−ve^ tongue tumor tissues and cell lines. Gel-shift assays showing NF-κB DNA binding activity in nuclear extracts (10 μg) from different grades of tongue tumor tissues including normal controls (A, B) and cell lines (C) using γ^32^P- ATP-radiolabeled oligonucleotide harbouring an NF-κB consensus sequence. Increasing NF-κB DNA binding activity was observed as the severity of tongue lesions progressed from normal to precancer to invasive cancer in both HPV16^+ve^ and HPV^−ve^ (A, B) patients. Both HPV16^+ve^ UPCI:SCC090 and HPV^−ve^ AW13516 cell lines (C) showed increased NF-κB DNA binding activity but relatively higher in HPV^−ve^ cells. (D) EMSA with labeled Oct-1 probe showed similar DNA binding activity in all grades of tongue tissues. Binding specificity was confirmed in a competition assay with a 100× molar exess addition of the specific cold competitor (NF-κB) probe and nonspecific competitor Oct-1 probe and checked by EMSA (E). Densitometric analysis of band intensities of NF-κB are indicated below each lane.

To investigate the structural and functional role of NF-κB during tongue carcinogenesis, we have analyzed the DNA binding activity of NF-κB and its family proteins in TSCC tissues and cell lines by gel shift assay. The constitutive activation of NF-κB as revealed by gradual increase in DNA binding activity of NF-κB with increasing severity of the tongue lesions was observed (see Figure 1A–1C). Interestingly, there was a clear difference in binding activity of NF-κB between HPV^+ve^ and HPV^−ve^ tumors; a little higher DNA binding was observed in HPV^−ve^ TSCC cases (Figure 1B). The specificity of NF-κB binding activity was confirmed by performing cold competition assays with 100× molar excess of homologous NF-κB and a heterologous Oct-1 as an internal control (Figure 1D, 1E).

**Figure 2:**
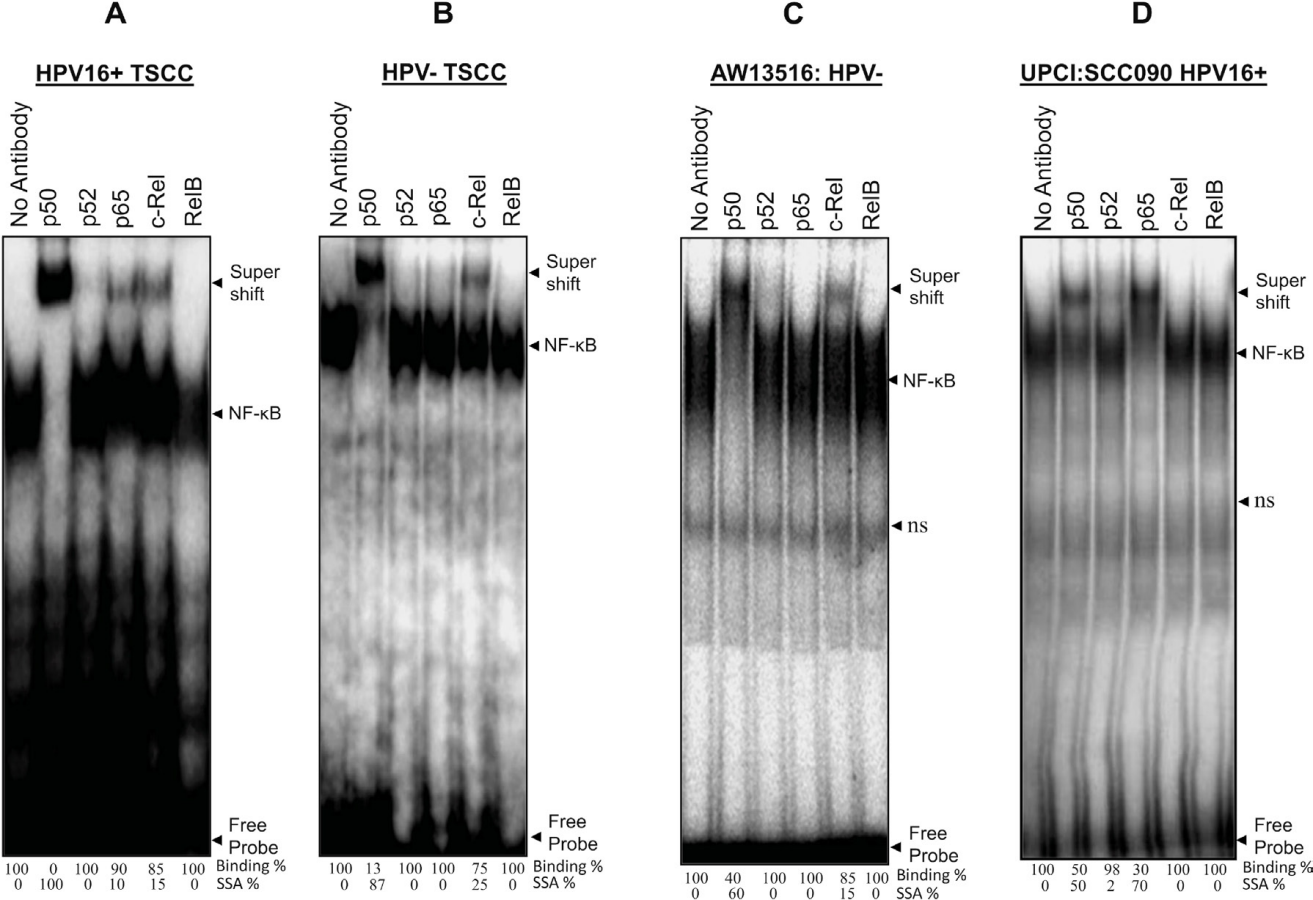
(**A**–**D**) Differential composition of NF-κB complex formation in HPV^+ve^ and HPV^−ve^ tongue tumor tissues and cell lines. Gel-supershift assays using specific antibodies against NF-κB proteins; p50, p52, p65, c-Rel & RelB. Panel A, B, C and D showing differential DNA binding activity of NF-κB subunits in HPV^+ve^ (A) and HPV^−ve^ (B) TSCC and cell lines; AW13516; HPV^−ve^ (C) and UPCI:SCC090; HPV16^+ve^ (D). In all panels (A–D) significantly a higher binding activity of p50 which forms the major part (~90%) of functional NF-κB complex. HPV^−ve^ TSCC (panel B) consistently shows a minor participation of c-Rel with p50 and while p65 selectively participates in HPV^+ve^ tumors (panel A). Lane No antibody for panel A–D, used as negative control. The intensities of super-shifted bands indicated and quantified in densitometric analysis. NS; non-specific binding.

To analyze the composition of functional NF-κB complex in presence and absence of HPV infection, gel supershift assays were performed using specific antibodies (Santacruz, USA) raised against all five NF-κB family proteins; p50, p52, p65, c-Rel and RelB. The gel supershift assay revealed a differential DNA binding pattern and difference in composition of NF-κB complex. p50 was the major DNA binding partner involved in the formation of functional NF-κB complex in both HPV^+/−ve^ tumors (Figure 2A, 2B) but majority of the HPV^−ve^ (27/36; 75%) and poorly differentiated tumors showed minor but consistent involvement of c-Rel (~15–25%) with p50 (~80–90%) in the functional NF-κB complex (Figure 2B and Table 1B). In contrast, the majority (11/14; 78.6%) of HPV^+ve^ and well differentiated tumors showed selective participation of p65 (~10–50%) in addition to p50-c-Rel (Figure 2A and Table 1B). No shift was observed for other NF-κB family proteins in both HPV^+/−ve^ tumors. In almost all tumors, more than 90% of supershifted bands were formed by p50/p50 homodimers with minor but consistent involvement of c-Rel in most of the higher grade tumors indicating a critical role of p50/c-Rel proteins in generation of functional NF-κB complex and transactivation during development of TSCC (Figure 2A, 2B, Table 1B). When we compared these findings with that of TSCC cell lines, p50 and c-Rel also form the functional NF-κB in HPV^−ve^ cells (AW13516) (Figure 2C) whereas HPV^+ve^ cells (UPCI: SCC090) showed regular participation of p65 along with p50 (Figure 2D); however, no involvement of c-Rel was found in the functional NF-κB complex formation.

**Table 1B:** Analysis of binding patterns of NF-κB family proteins in HPV16^+ve^ & HPV^−v^^e^ tongue cancer cases by band super shift assay

NF-κB family proteins	TSCC (*n* = 50)								*p*-values
	HPV16 positive TSCC (*n* = 14)				HPV negative TSCC (*n* = 36)				
	Nil -	Weak +	Moderate ++	Strong ++++	Nil -	Weak +	Moderate ++	Strong ++++	
**P50**	**-**	**1**	**4**	**9**	**3**	**4**	**5**	**24**	0.41 (ns)
**P52**	**14**	**-**	**-**	**-**	**36**	**-**	**-**	**-**	1.0 (ns)
**P65**	**3**	**2**	**9**	**-**	**36**	**-**	**-**	**-**	**0.0001**
**c-Rel**	**-**	**4**	**10**	**-**	**9**	**7**	**19**	**1**	0.35 (ns)
**RelB**	**14**	**-**	**-**	**-**	**36**	**-**	**-**	**-**	1.0 (ns)

^*^Data represent DNA binding activity of NF-κB family proteins in >90% of cases with minor changes in few cases. Arbitrary level of DNA binding in band supershift assay: nil/not detectable (−); Weak (+); Moderate (++); Strong (+++). *p-value* obtained by probability Fisher's (exact) test using Graph Pad Instat (6.0) comparing the DNA binding of NF-κB family members (nil + weak Vs moderate + strong) among HPV^+ve^ cancer Vs HPV^−ve^ cancer. *p-values* ≤ 0.05 are considered as statistically significant. ns = not significant.

### Expression of NF-κB family proteins increased as a function of severity of tongue lesions

**Figure 3:**
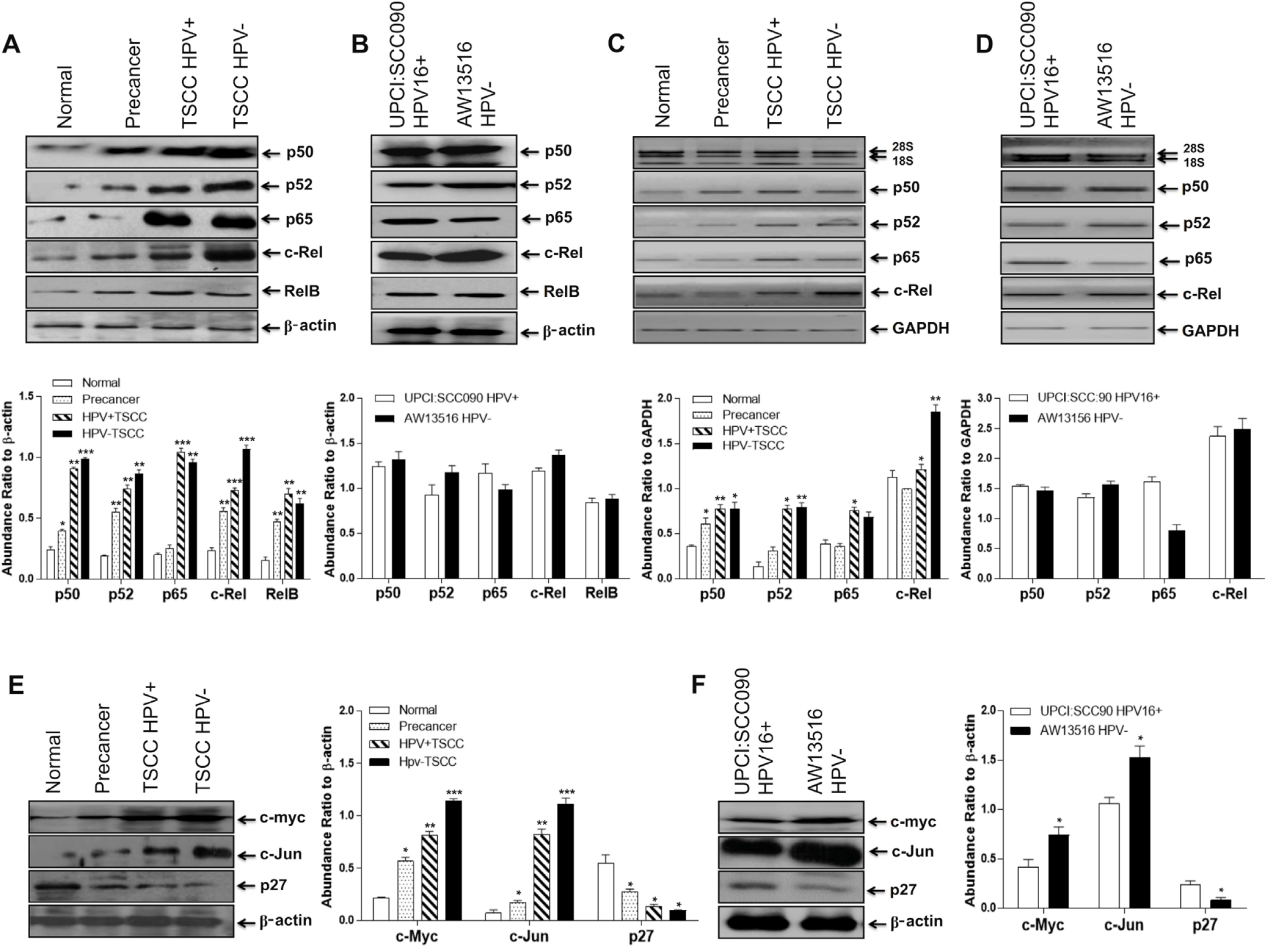
(**A**–**F**) Differential expression pattern of NF-κB proteins and their target genes in different grades of tongue tissues and cell lines. Representative immunoblots showing differentially overexpressed NF-κB proteins and their target genes (c-myc and c-Jun) in tongue tumor biopsies (A) and cell lines (B). 40 μg cellular protein from tongue tissues or cell lines were resolved on 10% SDS-PAGE, electrotransferred and probed with specific antibodies against all NF-κB family proteins (p50, p52, p65, c-Rel & RelB) and their target genes (c-myc, c-Jun and p27). To confirm equal protein loading, the membranes were stripped and re-probed for β-actin expression and quantitation of bands was performed by densitometric analysis showing in graphical panel of A, B. RT-PCR showing elevated mRNA expression profile of p50, p52, p65 & c-Rel transcripts in tongue tumor tissues (C) and tumor cell lines (D). GAPDH gene was used as internal loading control (lower panel of C & D). Panel E, F; show differential expression of c-myc, c-Jun and p27 genes in HPV^+ve^ and HPV^−ve^ tongue tumor tissues (E) and cell lines (F). β-actin gene was used as an internal control (lower panels of E, F). Significant (*p <* 0.05) when compared with control vs. cases and HPV^+ve^ vs. HPV^−ve^ cell lines. The data in bar diagram are expressed as the mean (±SEM or ±SD) of three independent experiments. ^*^p < 0.05 & ^**^p < 0.01, ^***^p < 0.001 & ^***^p < 0.0001.

To determine the pattern and level of expression of NF-κB family proteins during TSCC, immunoblotting experiments were performed with cellular proteins extracted from tongue tumors and adjacent controls and cell lines. As illustrated in the Figure 3A, significantly a higher expression of NF-κB family proteins; p50 (34/50, 68%; *p* = 0.0002), p52 (29/50, 58%; *p* = 0.0001), p65 (30/50, 60%; *p* = 0.0001) and c-Rel (37/50, 74%; *p* = 0.0001) was observed in majority of tongue cancer cases as compared to adjacent normal controls (Figure 3A and Table 1C). On the other hand, precancer cases showed moderate expression of p50 (10/20, 50%), p52 (11/20, 55%) and c-Rel (11/20, 55%; *p* = 0.008) proteins (Figure 3A and Table 1C). Immunoblotting data well corroborated with the results obtained from band supershift assays (Figure 2A, 2B, Table 1B). RelB (25/50, 50%; *p* = 0.03) showed an inconsistent pattern of expression.

**Table 1C:** Expression of NF-κB family members & their target genes products in tongue precancer, cancer and normal control cases

Target proteins (↓)	Adjacent normal controls (*n* = 30)				Precancer (*n* = 20)				TSCC (*n* = 50)				*p-value*
Expression level (→)	Nil (−)	Weak (+)	Moderate (++)	Strong (++++)	Nil (−)	Weak (+)	Moderate (++)	Strong (++++)	Nil (−)	Weak (+)	Moderate (++)	Strong (++++)	
***P50***	**2**	**9**	**14**	**5**	**2**	**4**	**10**	**4**	**-**	**2**	**14**	**34**	0.7^1^, **0.0002^2^, 0.005^3^**
***Cases in statistical group (%)***	11 (36.6%)		19 (63.4%)		6 (30%)		14 (70%)		2 (4%)		48 (96%)		
***P52***	**5**	**10**	**12**	**3**	**1**	**5**	**11**	**3**	**-**	**1**	**20**	**29**	0.2^1^**, 0.0001^2^**, **0.001^3^**
***Cases in statistical group (%)***	15 (50%)		15 (50%)		6 (30%)		14 (70%)		1 (2%)		49 (98%)		
***P65***	**7**	**12**	**11**	**-**	**3**	**5**	**10**	**2**	**-**	**3**	**17**	**30**	0.1^1^**, 0.0001^2^, 0.001^3^**
***Cases in statistical group (%)***	19 (63.3%)		11 (36.7%)		8 (40%)		12 (60%)		3 (6%)		47 (94%)		
***c-Rel***	2	14	10	4	**-**	**3**	**11**	**6**	**-**	**-**	**13**	**37**	**0.008^1^, 0.0001^2^, 0.02^3^**
***Cases in statistical group (%)***	16 (53.3%)		14 (46.7%)		**3 (15%)**		**17 (85%)**		**0 (0%)**		**50 (100%)**		
***RelB***	**2**	**5**	**14**	**9**	**1**	**4**	**12**	**3**	**1**	**2**	**22**	**25**	1.00^1^, **0.03^2^, 0.03^3^**
***Cases in statistical group (%)***	7 (23.3%)		23 (76.7%)		5 (25%)		15 (75%)		3 (6%)		47 (94%)		
***c-myc***	**1**	**8**	**15**	**6**	**-**	**2**	**11**	**7**	**-**	**-**	**12**	**38**	0.1^1^, **0.0001^2^**, 0.07^1^
***Cases in statistical group (%)***	9 (30%)		21 (70%)		2 (30%)		18 (70%)		0 (0%)		50 (100%)		
***P27***	**2**	**5**	**13**	**10**	**2**	**5**	**7**	**6**	**9**	**20**	**18**	**3**	0.5^1^, **0.002^2^**, 0.1^3^
***Cases in statistical group (%)***	7 (23.3%)		23 (76.7%)		7 (35%)		13 (65%)		29 (58%)		21 (42%)		

Arbitrary level of expression in immunoblotting: Nil/not detectable (−); Weak (+); Moderate (++); Strong (+++). *p-value* obtained by probability Fisher's (exact) test using Graph Pad Instat 6.0 comparing the expression of proteins (nil + weak Vs moderate + strong) among ^1^Control Vs Precancer, ^2^Control Vs Cancer, ^3^Precancer Vs cancer, ^4^HPV ^+ve^ cancer Vs HPV^−ve^ cancer. *p-values* ≤ 0.05 are considered as statistically significant. ns = not significant.

When, we correlated expression pattern of NF-κB family proteins between HPV^+ve^ and HPV^−ve^ tongue cancer cases, a slightly higher expression of p50, p52 and c-Rel proteins was observed mainly in HPV^−ve^ cases as compared to HPV16^+ve^ TSCCs that selectively overexpressed p65 protein (Figure 3A and Table 1C). A good correlation has been observed between the increasing expression pattern of p50, p52, p65 and c-Rel and the severity of tongue cancer lesions as they progressed from normal to precancer to malignant phenotype. TSCC cell lines also showed similar strong expression of p50, p52, p65, c-Rel including RelB in both HPV^+/−ve^ tongue cancer cells, however, the extent of c-Rel expression was distinctly higher in HPV^−ve^ cells (Figure 3B).

### Overexpression of NF-κB family gene transcripts during TSCC

Since most of the TSCCs displayed generalized increase in the expression of NF-κB proteins, we next examined their gene transcripts in total RNA isolated from tongue tissue biopsies of cancer, precancer and normal controls including tongue cancer cell lines by NF-κB family gene-specific qRT-PCR. The majority of tongue cancer cases showed higher level of expression of p50, p65 and c-Rel transcripts as compared to precancer and normal controls and the level of transcripts increased gradually as the lesions progressed to malignant phenotype (Figure 3C). These results well corroborated with the results of immunoblotting (Figure 3A, 3B) and gel supershift assays (Figure 2A, 2B). Similar results were recorded in tongue cancer cell lines where the level of p65 transcript was specifically higher in HPV^+ve^ cells (Figure 3D).

### c-Rel activates c-myc and c-Jun but downregulates p27 expression

Since c-Rel, is often found activated and overexpressed in TSCCs, we examined the expression of its target genes; c-myc, c-Jun and p27 by immunoblotting. Enhanced expression of c-Rel led to activation of its downstream target genes such as c-myc, c-Jun and CDK but down-regulation of p27 leading to enhanced cell proliferation and aggressive tumor progression. The results revealed significantly a higher expression of c-myc (38/50, 76%; *p* = 0.0001) and c-Jun (30/50, 60%; *p* = 0.0001) in majority of tumors as compared to precancer and controls (Figure 3E, Table 1C). Interestingly, the magnitude of overexpression of these proteins was relatively higher in HPV^−ve^ tumors (Figure 3E, Table 1D) though; not statistically significant. In contrast, majority (29/50, 58%; *p* = 0.002) of tumors showed significantly a low or undetectable expression of p27 which was relatively higher in normal controls (Figure 3E). It is interesting to note here that along with c-myc and AP-1/c-Jun, Fra-2 was also highly upregulated in both TSCC tissues and cell lines and their levels were highly pronounced in HPV^−ve^ cells, whereas, p27 obviously showed low level of expression in both HPV^+/−ve^ cells (Figure 3F).

**Table 1D:** Expression of NF-κB family members between HPV16^+ve^ and HPV^−ve^ tongue cancer cases

*Target proteins* (↓)	Total TSCC (*n* = 50)								*p-value*
	*HPV16 positive TSCC (n = 14)*				*HPV negative TSCC (n = 36)*				
Expression level (→)	Nil -	Weak +	Moderate ++	Strong ++++	Nil -	Weak +	Moderate ++	Strong ++++	
***P50***	**-**	**1**	**2**	**11**	**-**	**1**	**12**	**23**	0.4^4^ (ns)
***Cases in statistical group (%)***	**1 (7.1%)**		**13 (92.9%)**		**1 (2.7%)**		**35 (91.3%)**		
***P52*** **-**		**1**	**3**	**10**	**-**	**-**	**17**	**19**	0.2^4^ (ns)
***Cases in statistical group (%)***	**1 (7.1%)**		**13 (92.9%)**		**0 (0%)**		**36 (100%)**		
***P65***	**-**	**1**	**2**	**11**	**-**	**2**	**15**	**19**	1.00^4^ (ns)
***Cases in statistical group (%)***	**1 (7.1%)**		**13 (92.9%)**		**2 (5.5%)**		**34 (94.5%)**		
***c-Rel***	**-**	**-**	**4**	**10**	**-**	**-**	**9**	**27**	1.00^4^ (ns)
***Cases in statistical group (%)***	**0 (0%)**		**14 (100%)**		**0 (0%)**		**36 (100%)**		
***RelB***	**-**	**1**	**6**	**7**	**1**	**1**	**16**	**18**	1.00^4^ (ns)
***Cases in statistical group (%)***	**1 (7.1%)**		**13 (92.9%)**		**2 (5.5%)**		**34 (94.5%)**		
***c-myc***	**-**	**-**	**8**	**6**	**-**	**-**	**4**	**32**	1.00^4^ (ns)
***Cases in statistical group (%)***	**0 (0%)**		**14 (100%)**		**0 (0%)**		**36 (100%)**		
***P27***	**2**	**4**	**5**	**3**	**7**	**16**	**13**	**-**	0.14^4^ (ns)
***Cases in statistical group (%)***	**6 (42.8%)**		**8 (51.2%)**		**23 (63.8%)**		**13 (36.2%)**		

Arbitrary level of expression in immunoblotting: Nil/not detectable (−); Weak (+); Moderate (++); Strong (+++). *p-value* obtained by probability Fisher's (exact) test using Graph Pad Instat 6.0 comparing the expression of proteins (nil + weak Vs moderate + strong) among HPV^+ve^ cancer Vs HPV^−ve^ cancer. *p-values* ≤ 0.05 are considered as statistically significant. ns = not significant.

### c-Rel siRNA selectively downregulates NF-κB/c-Rel expression in TSCC cells

**Figure 4:**
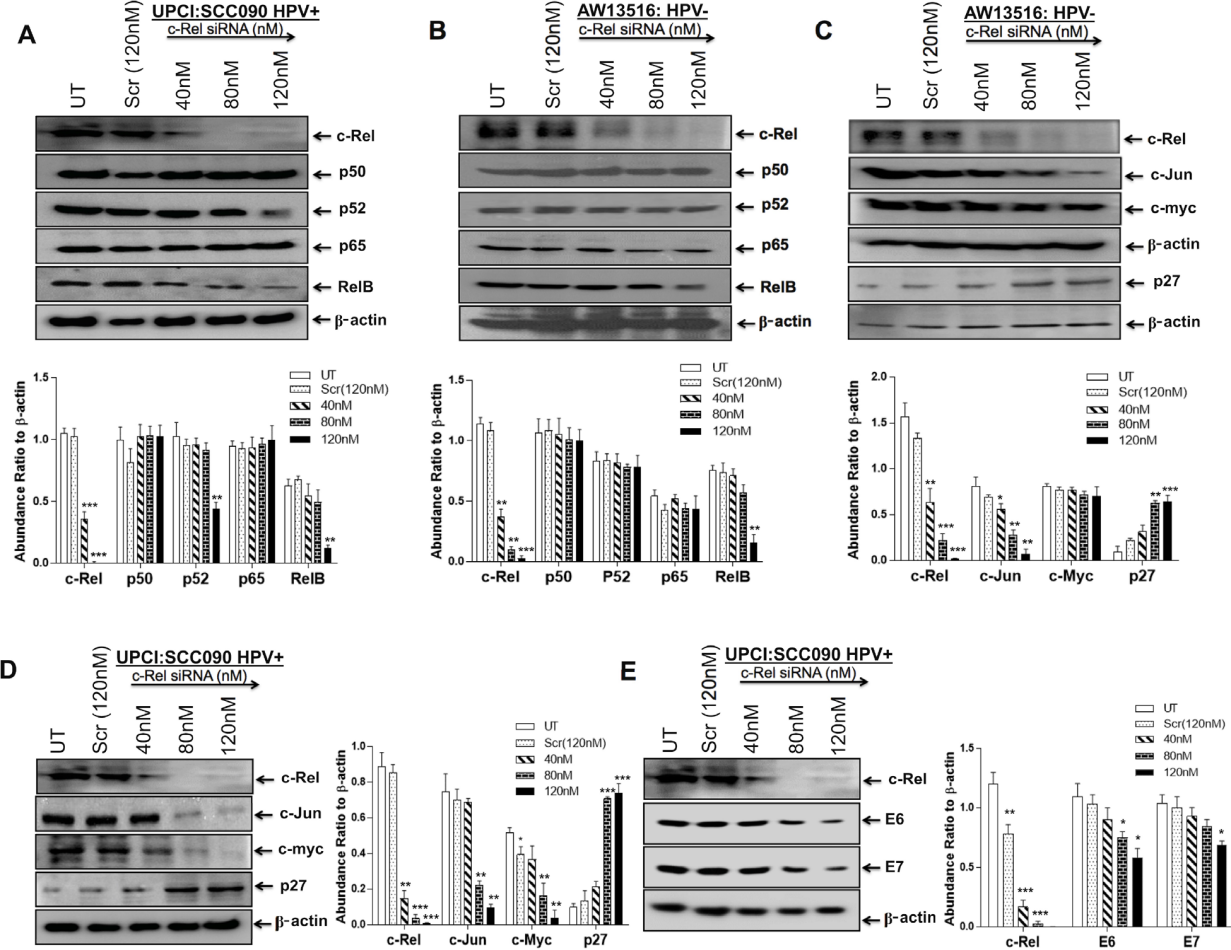
(**A**–**E**) Knocking down of c-Rel by specific siRNA in HPV^+ve^ and HPV^−ve^ TSCC cells. Representative immunoblots (Panel A, B) showing complete inhibition of c-Rel expression at the concentration of 80 nM–120 nM in both UPCI:SCC090; HPV16^+ve^ (A) and AW13516; HPV^−ve^ cells (B). 40 μg cellular proteins isolated from both the cell lines following treatment with c-Rel-siRNA (40 nM–120 nM) and scrambled siRNA (120 nM) as control for 48 hours were examined for its effects on c-Rel and other NF-κB family proteins (A and B) and also on their targets; c-myc, c-Jun and p27 (C, D) and HPV16 E6/E7 oncogenes (E). To ensure equal loading of protein, β-actin was used as an internal control. Quantitation of bands was performed by densitometric analysis. Significant (*p <* 0.05) when compared with untreated control vs. siRNA treated group. The data in corresponding bar diagram are expressed as the mean ± SD of three independent experiments. ^*^p < 0.05, ^**^p < 0.01 & ^***^p < 0.001.

Our results demonstrate consistent involvement of c-Rel in the formation of functional NF-κB complex, DNA binding activity and transactivation. To explore it further, in the next part of our investigation, we examined the effect of *in-vitro* silencing of c-Rel in both the HPV^+ve^ and HPV^−ve^ tongue cancer cells using specific commercially synthesized small interfering RNA (siRNA) raised against c-Rel. The cells were transiently transfected with a pool of c-Rel-specific siRNA at a concentration of 40 nM, 80 nM and 120 nM for 48 hours. Western blot analysis was performed to check efficiency of c-Rel knockdown and its effect on the expression of NF-κB family proteins. Cells transfected with scrambled siRNA (120 nM) served as control. Transfection of TSCC cells by increasing concentration (40 nM–120 nM) of c-Rel siRNA demonstrated a dose-dependent decline in the expression of c-Rel protein while its expression remained unaltered in scrambled siRNA treated cells (Figure 4A, 4B). The c-Rel suppression was observed even at concentration as low as 40 nM and it was completely inhibited at 120 nM in both HPV^+/−ve^ cells (Figure 4A, 4B). This was accompanied by reduction in RelB expression however, p50, p52 and p65 expression remained unaffected (Figure 4A, 4B). The effect of c-Rel siRNA on cell proliferation was examined microscopically using trypan blue dye exclusion assay. As depicted (Supplementary Figure 2A, 2B), the cultures showed about 30–40% reduction in cell proliferation after c-Rel-siRNA treatment as compared to untreated or scrambled-siRNA treated controls (Supplementary Figure 2A, 2B).

### Inhibition of c-Rel down-regulates c-myc, c-Jun, Fra-2 and HPV16 E6/E7 but induces p27Kip1 expression in TSCC cells

**Figure 5:**
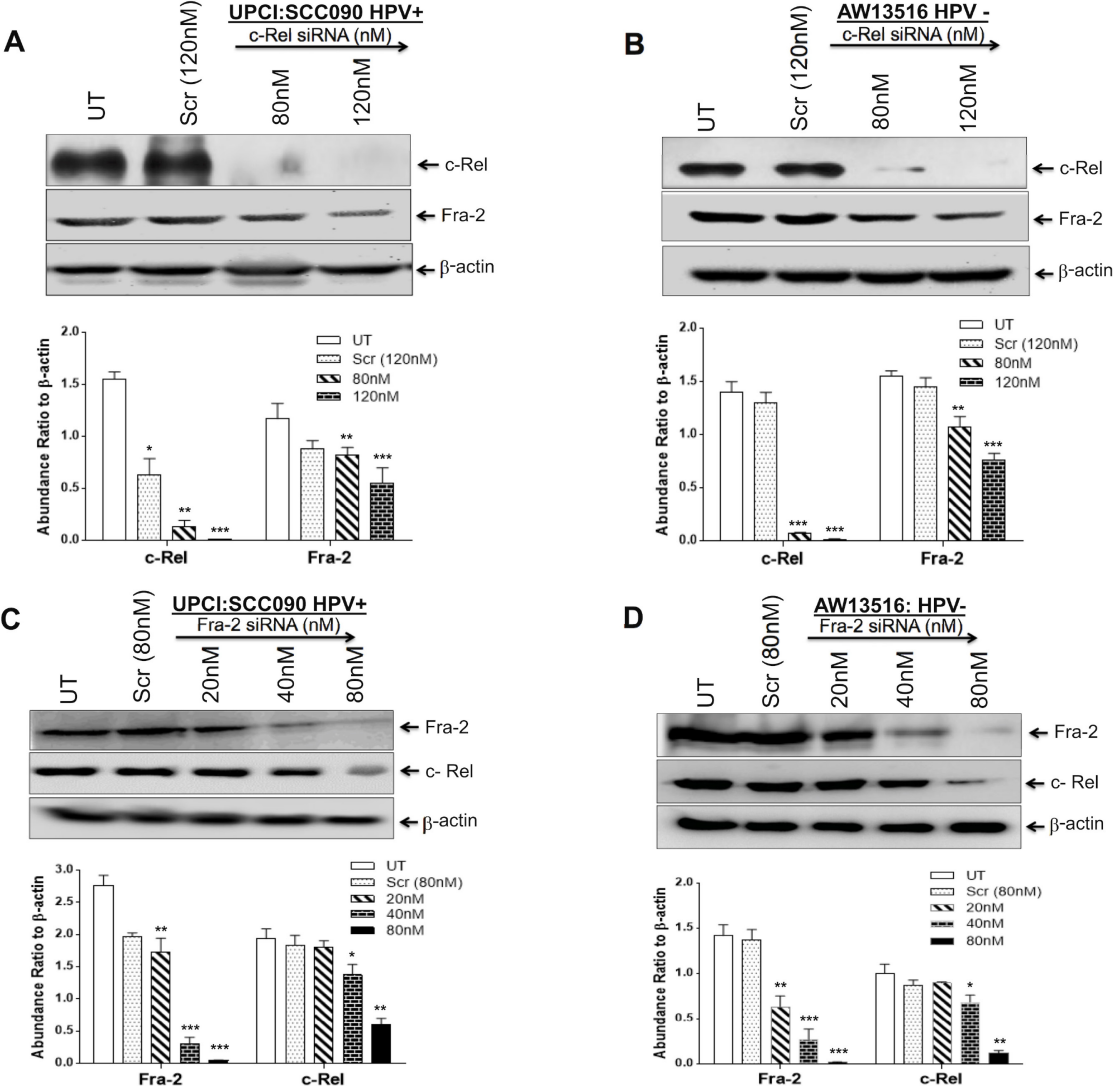
(**A**–**D**) AP-1/Fra-2 and NF-κB/c-Rel specific silencing altered the expression of c-Rel and Fra-2 in both HPV^+ve^ and HPV^−ve^ TSCC cells. siRNA knockdown efficiencies were analysed by western blotting (A–D upper panel). c-Rel siRNA at 120 nm of concentration reduced the expression of Fra-2 in both HPV^+ve^ (A) and HPV^−ve^ (B) TSCC cells. C, D show reduction in the expression of c-Rel in both HPV^+ve^ (C) and HPV^−ve^ (D) TSCC cells following 80 nM of Fra-2 siRNA. Equal loading of protein was checked by using β-actin as internal control. Quantitation of bands was performed by densitometric analysis and are shown in corresponding bar diagrams. Significant (*p <* 0.05) when compared with untreated control vs. treated group. The data in bar diagram are expressed as the mean ± SD of three independent experiments. ^*^p < 0.05 & ^**^p < 0.01.

To investigate whether silencing of c-Rel affects the expression of downstream genes c-myc, c-Jun, p27 and HPV16 E6/E7 involved in TSCC carcinogenesis, we transfected TSCC cells (UPCI:SCC090 and AW13516) with different concentration of c-Rel siRNA for 48 hours. The transfection efficiency of c-Rel was analysed by western blotting. The maximum (~95%) transfection efficiency of c-Rel was achievable at 120 nM in both HPV^+ve/−ve^ cell lines (Figure 4C–4E, upper panel). The results of c-Rel knockdown showed inhibition of c-Rel was accompanied by reduced expression of c-myc and c-Jun (Figure 4C, 4D) which were more prominent in HPV^+ve^ UPCI:SCC090 cells (Figure 4D). An inverse effect was observed in the expression of p27 protein that was markedly increased with increasing concentrations of c-Rel siRNA irrespective of the HPV status (Figure 4C, 4D). Following c-Rel siRNA treatment, the expression of HPV16 E6/E7 was also reduced but only at the highest concentration (Figure 4E). Interestingly enough, when we checked the effect of c-Rel siRNA on AP-1/Fra-2 which was shown to be highly overexpressed in TSCCs [6], a significant reduction in Fra-2 expression was observed in both HPV^+/−ve^ cells (Figure 5A, 5B). It indicates that there exists a cross-talk between Fra-2 and c-Rel during tongue tumorigenesis.

### Fra-2 siRNA downregulates c-Rel indicating a possible cross-talk between the two genes contributing to aggressive TSCC

We have recently demonstrated that selective participation and overexpression of Fra-2 in presence of c-Jun is associated with aggressive tumor phenotype of HPV^−ve^ TSCCs and its selective silencing by Fra-2-siRNA suppressed tumor metastasis [6]. This finding prompted us to re-examine the extent of contribution of Fra-2 in the functional activity of c-Rel in inducing aggressive tongue tumorigenesis. Interestingly, we found a remarkable inhibitory effect of Fra-2-siRNA on the expression of c-Rel in TSCC cells. As shown in Figure 5C, 5D, Fra-2-siRNA at 80 nM concentration dramatically reduced the level of c-Rel expression in TSCC cells. It shows a clear cross-talk between NF-κB/c-Rel and AP-1/Fra-2. The knockdown efficiency determined by western blotting demonstrated ~90% suppression of c-Rel and Fra-2 in both HPV^+ve/−ve^ cell lines (Figure 5A–5D, upper panel).

### Knocking down of c-Rel reduced invasive and migration property of tongue cancer cells

**Figure 6:**
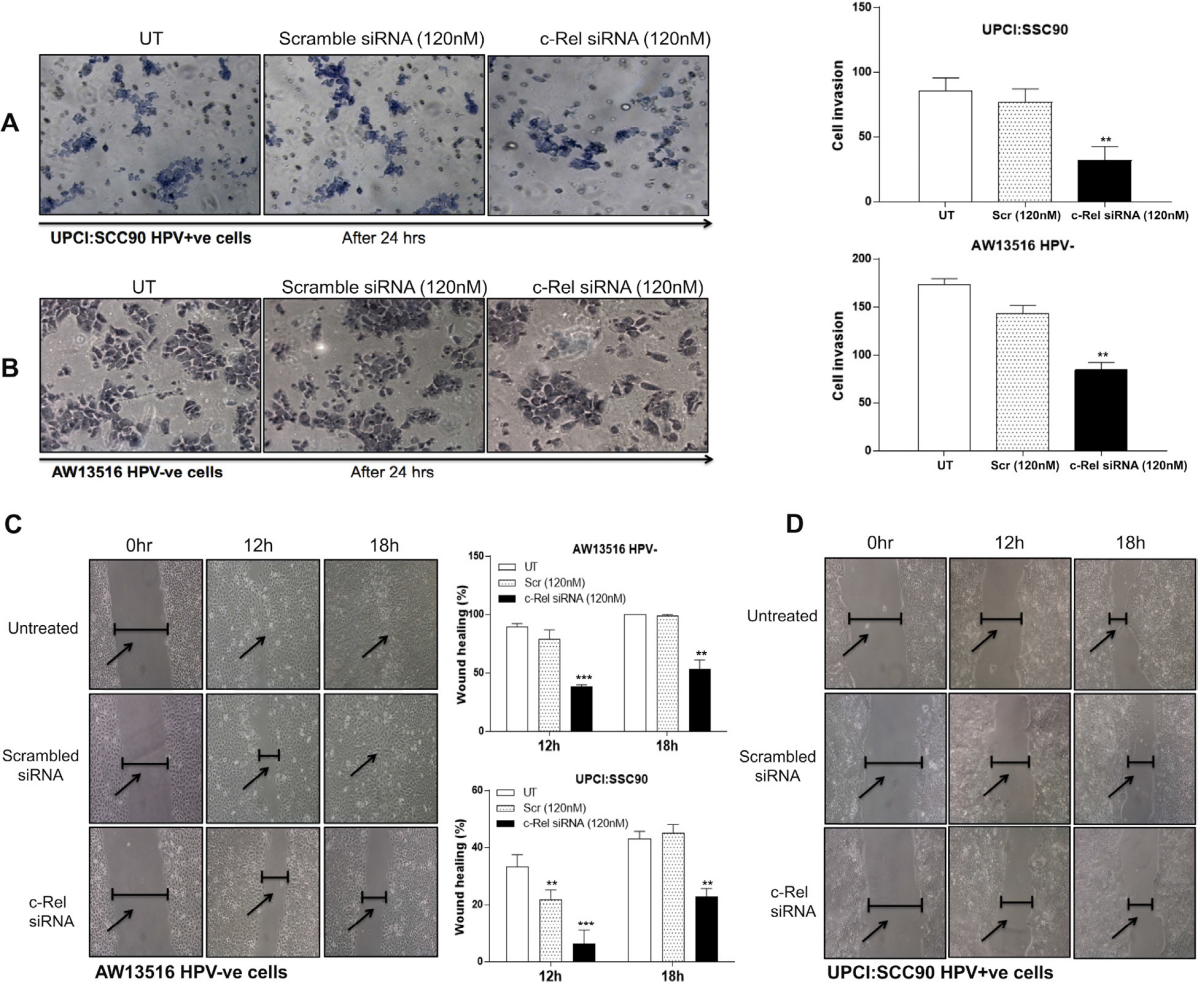
(**A**–**D**) NF-κB/c-Rel knockdown reduces the invasive and migration ability of HPV^+ve^ and HPV^−ve^ TSCC cells. c-Rel silencing by specific siRNA leads to alteration in invasion property in both HPV^+ve^ (A) and HPV^−ve^ (B) TSCC cells. A matrigel transwell invasion assay was used to evaluate the migration ability of untreated, scrambled siRNA and c-Rel-siRNA treated TSCC cells. Equal number of cells (1 × 10^5^) was seeded in the matrigel coated upper chamber. After 24 hours of incubation at 37^°^ C and 5% CO_2_, the cells that invaded through the membrane were fixed and stained with Giemsa, and images were captured using inverted microscope. Representative fields of view for each well are shown (A, B). Cell invasion was quantified by counting cells from six random fields. Panel C, D: Scratch assay after c-Rel silencing demonstrated that cell migration was dramatically decreased in experimental groups after c-Rel siRNA transfection compared with untreated and scrambled treated control groups. Inverted microscope images of wound closure at 0 hour, 12 hours and 18 hours in AW13516 (C) and UPCI:SCC090 (D) cells are indicated. Migration rate was calculated by image J software. The data in corresponding bar diagram are expressed as the mean ± SD of three independent experiments. ^*^p < 0.05, ^**^p < 0.01, ^***^p < 0.001 & ^****^p < 0.0001.

To examine the efficiency of c-Rel silencing on the invasive properties of both HPV^+/−ve^ TSCC cells, we performed matrigel invasion assay (Figure 6A, 6B) using standard procedure [6]. The results demonstrated that the invasion of both HPV^+ve^ and HPV^−ve^ cells was significantly reduced after c-Rel silencing when compared with invasion of untreated or scrambled siRNA treated cells (Figure 6A, 6B). Interestingly, c-Rel silencing at 120 nM of concentration was more effective in HPV^+ve^ (UPCI:SCC090) (Figure 6A) cells as compared with HPV^−ve^ (AW13516) cells (Figure 6B). Quantitation of the invading HPV^+ve^ cells showed only 37% (±32 cells, *p* = 0.001) when compared to untreated (±85.6 cells; 100%) or scrambled siRNA treated cells (±77 cells; 89.9%) (Figure 6A). But HPV^−ve^ cells showed 48.5% (±84.3 cells, *p* = 0.008) invasion after c-Rel siRNA treatment as compared to untreated (±173.6 cells, 100%) or scrambled treated groups (±143.6 cells; 82.6%) (Figure 6B). The untreated groups served as negative control and scrambled siRNA treated was considered as treated controls for both the cell lines.

To further demonstrate that the change in migration ability to accelerate the closure of a wound after c-Rel inhibition in TSCC cells, wound assay was performed after silencing of c-Rel in both AW13516 (HPV^−ve^) and UPCI:SCC090 (HPV16^+ve^) cells. The results revealed that c-Rel silencing for 18 hours in HPV^−ve^ cells showed ~53% migration when compared to untreated or scrambled treated control cells that showed ~100% and 99% migration respectively (Figure 6C). Interestingly, c-Rel knockdown in HPV16^+ve^ cells showed only ~22% migration at 18 hours while untreated or scrambled treated cells showed ~45% and ~43% migration rate respectively (Figure 6D). These results together well correlate with the significant reduction in cell invasion and migration after knocking down c-Rel in both HPV^+/−ve^ tongue cancer cells.

### String analysis of NF-κB indicates intricate interactive network and signalling pathways of c-Rel

**Figure 7:**
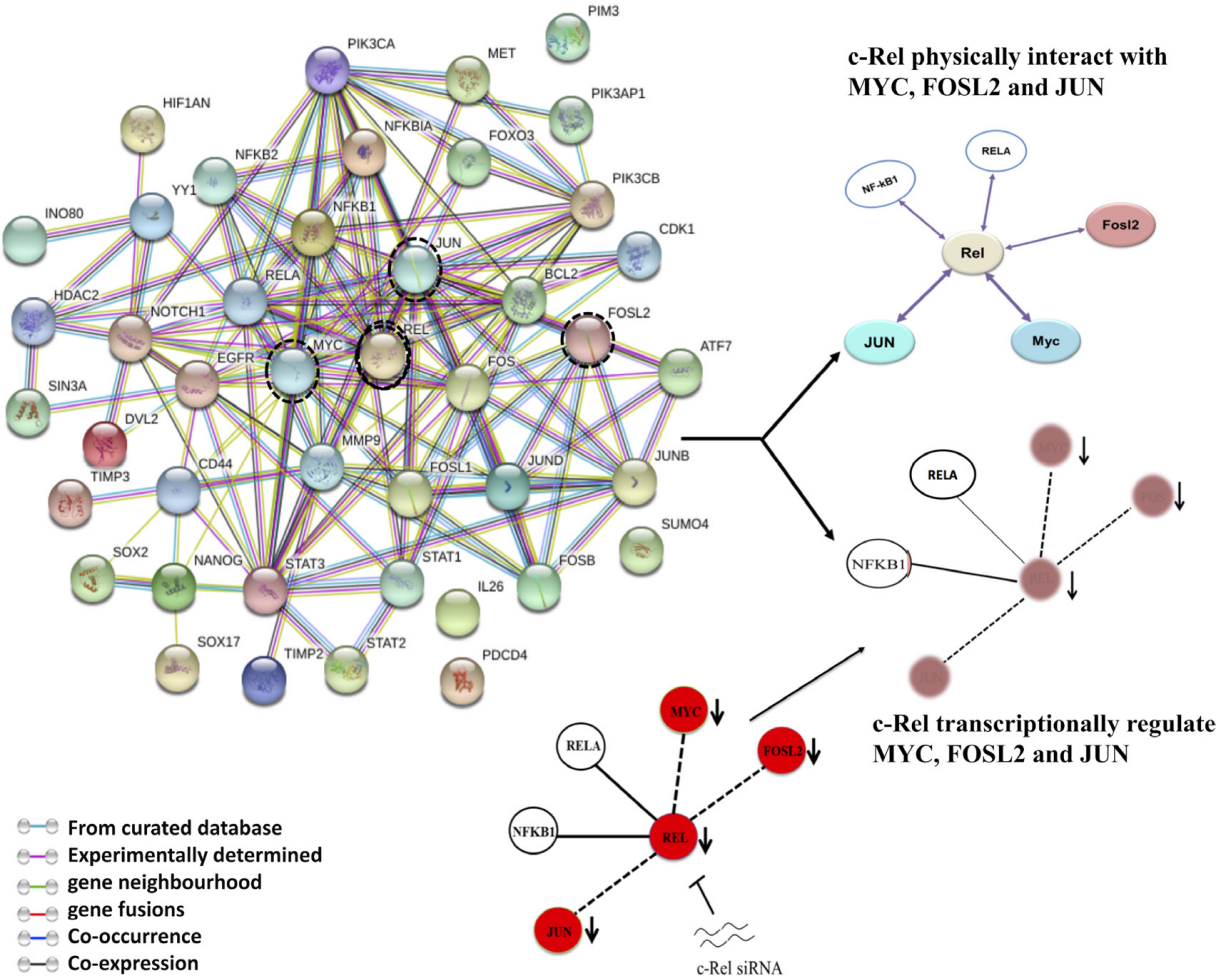
String network of NF-κB showing interactive pathways of c-Rel A cancer-associated protein-protein interaction (PPI) network of NF-κB was constructed using STRING server on the basis of highest confidence (score > 0.7). c-Rel knockdown reduces the levels of p52, RelB, c-Jun, c-myc and Fra-2 as shown in the results indicating thereby that c-Rel transcriptionally regulate these genes. c-Rel also physically interacts with the products of c-Jun, c-myc, Fra-2 indicating c-Rel forms both transcriptional network as well as protein-protein interaction network.

We used search tool for the retrieval of interacting genes (STRING) server analysis to identify possible physical and functional network of NF-κB, AP-1 and their downstream target genes and signalling pathways and it revealed involvement of high-degree (combined score of 0.7) hub genes in the local network (Figure 7). We found that inhibition of c-Rel by c-Rel siRNA reduces the expression level of p52, RelB, c-myc, c-Jun and Fra-2 as shown in the results indicating thereby that c-Rel transcriptionally regulate these genes. In addition, c-Rel also physically interacts with the products of c-Jun, c-myc, Fra-2. It indicates c-Rel forms a transcriptional network (Figures 4C–4E and 5A–5D) as well as protein-protein interaction network (Figure 7). A number of NF-κB subunits physically interact with other signalling molecules, as indicated by STRING pathway analysis (Figure 7), include those genes whose expression has been altered after c-Rel knockdown, suggesting the fact that c-Rel transcriptionally regulates numerous essential genes that might play a critical role during tongue carcinogenesis.

## DISCUSSION

Present study examines the biological as well as clinical consequences of HPV infection and raises question as to how NF-κB plays a role in the aggressive tongue tumorigenesis. It also raises question whether there exists patho-physiological differences between HPV^+ve^ and HPV^−ve^ tumors contributing towards differential tumorigenicity and treatment outcome. Epidemiologic studies [4, 6, 25–28] including recent mutational landscape analysis of head and neck carcinoma have demonstrated a significant difference between HPV^+ve^ and HPV^−ve^ tumors [29]. The overall mutation rate was found to be almost two times higher in highly aggressive HPV^−ve^ tumors than that of HPV^+ve^ HNSCCs [30–32]. It is intriguing to find that the majority (94.4%) of HPV^−ve^ patients were tobacco smokers/chewers and/or alcohol users while HPV infection was found mainly in non-tobacco users (75%). It indicates that tobacco can inhibit HPV persistence in the oral cavity rather smoking protects from HPV infection possibly because of toxic effects and oxidative stress created by nicotine and/or alcohol. This is clear from our results that 8 (80%) out of 10 female TSCC patients were HPV^+ve^ and 75% (6/8) of them were non-smokers. In contrast, out of 40 male TSCC patients, all were tobacco smokers but only 6 (15%; 6/40) were HPV^+ve^ and 85% were HPV^−ve^ (Table 1A). Thus, there is a distinct difference between HPV^+ve^ and HPV^−ve^ tumors, HPV^−ve^ or tobacco associated tumors being both clinically and genomically at greater risk than the HPV^+ve^ for aggressive tumorigenesis and worst prognosis [33].

Analysis of HPV and their genotyping demonstrated 28% (14/50) exclusive prevalence (100%) of HR-HPV type 16, the majority (78.6%) of which were found in well differentiated tumors. In oral cancer, we were the first to demonstrate that well differentiated tumors induced by HPV infection as revealed by predominance of well stained koilocytes showed better prognosis when treated [12]. We also demonstrated that HPV infection promotes selective overexpression and regular participation of p65 in functional NF-κB complex formation leading to transactivation of p16, p21 and p27 genes involved in cell proliferation and differentiation [12], which has been later confirmed by several authors in HNSCCs [4, 6, 25–28].

Since infection, inflammation and cancer are intimately linked, the pro-inflammatory transcription factor, NF-κB is always constitutively activated and overexpressed in almost all cancers [12, 15, 19, 21, 22, 34]. In TSCC, NF-κB also show high DNA binding activity and differential overexpression of NF-κB proteins in majority of cases. We observed a slightly lower DNA binding activity of NF-κB in HPV16^+ve^ TSCCs. Gel supershift assays revealed p50 as the major DNA binding partner (~90%) in both HPV^+ve^ and HPV^−ve^ tumors and a small but distinct participation of c-Rel (~15–25%) was found in majority of HPV^−ve^ tumors. These HPV^−ve^ TSCCs showed either p50-p50 homo-dimer or heterodimer of p50-c-Rel (41/50; 82%) in the formation of functional NF-κΒ complex mainly in advanced stage poorly or moderately differentiated tumors. This is well corroborated with the western blotting results showing significantly higher expression of c-Rel in HPV-negative tumors and cell lines (Figure 3A, 3B). Overexpression of p50 and c-Rel proteins have been reported in variety of solid tumors and hematopoietic cancer [12, 15, 21, 35, 36]. It implies that these two proteins have the potential to induce aggressive tumorigenic phenotype and metastasis as reported by other authors [21–23, 37, 38]. c-Rel was also shown to be highly overexpressed in aggressive breast cancer [21], leukemias/lymphomas and in pancreatic ductal adenocarcinomas [22]. Together these findings strongly support activation of NF-κΒ with selective participation of c-Rel with p50 may play a critical role in inducing aggressive tumorigenesis specifically in HPV^−ve^ and poorly differentiated TSCC patients with tobacco habits who show worst prognosis.

Interestingly, HPV16^+ve^ TSCCs and cell lines (UPCI:SCC090) showed selective participation of p65 along with p50-c-Rel complex in majority (78.6%) of well differentiated tumors. Only three (21.4%) HPV16^+ve^ TSCC cases showed composition of p50-c-Rel but they were poorly or moderately differentiated tumors. It is therefore suggested that the involvement of p65 in NF-κB complex appears to be induced by HPV which is responsible for well differentiation of TSCC [12]. The minor but selective participation of c-Rel in DNA binding activity and its higher expression at mRNA and protein level in majority of TSCCs are indicative of its critical role in aggressiveness of the disease even it may not involve in DNA binding activity [12]. Furthermore, since cytokines or chemokines such as monocyte chemoattractant protein-1 (MCP-1) is differentially regulated in HPV16 positive cervical cancer, it would be highly interesting to look for expression of these chemokines being affected by altered NF-κB composition between HPV^+ve^ and HPV^−ve^ TSCCs [39, 40].

We also observed higher expression of c-myc and c-Jun which in conjunction with NF-κB proteins are known to play critical role in cellular transformation and metastasis [41–44]. The promoter region of NF-κB/c-Rel contains c-Jun and c-myc binding sites and a direct evidence has been provided for the critical role of c-Rel in aggressive breast carcinogenesis. These authors generated transgenic mice that overexpressed c-Rel in the mammary gland and more than 30% of these mice developed one or more mammary tumors. Thus, activation of c-Rel appears to induce expression of cyclin D1, c-Myc, and Bcl-xL that promote tumor growth.

Recently, we have shown overexpression and selective DNA binding activity of c-Jun with Fra-2 induced poor differentiation, aggressive tumor behaviour and metastasis specifically in HPV^−ve^ TSCCs [6]. c-Jun is known to involve in aggressive tumor phenotype and metastasis in many cancers [43, 45, 46] and c-myc is also shown to increase the expression of Cyclin D1 and decrease expression of p21, p27 and p53 leading to proliferative advantage of cancer cells [47–49]. It has been shown that c-Rel can induce activation of c-myc, c-Jun, CDKs and decrease p27Kip1, causing increased cell proliferation and aggressive tumor phenotype [49–51]. Our cell invasion and migration assays demonstrated that c-Rel silencing can remarkably reduced invasion and migration capability of both HPV^+ve^ and HPV^−ve^ tongue cancer cells (Figure 6A–6D). These results together indicate potential role of c-Rel in inducing invasion and metastasis.

**Figure 8:**
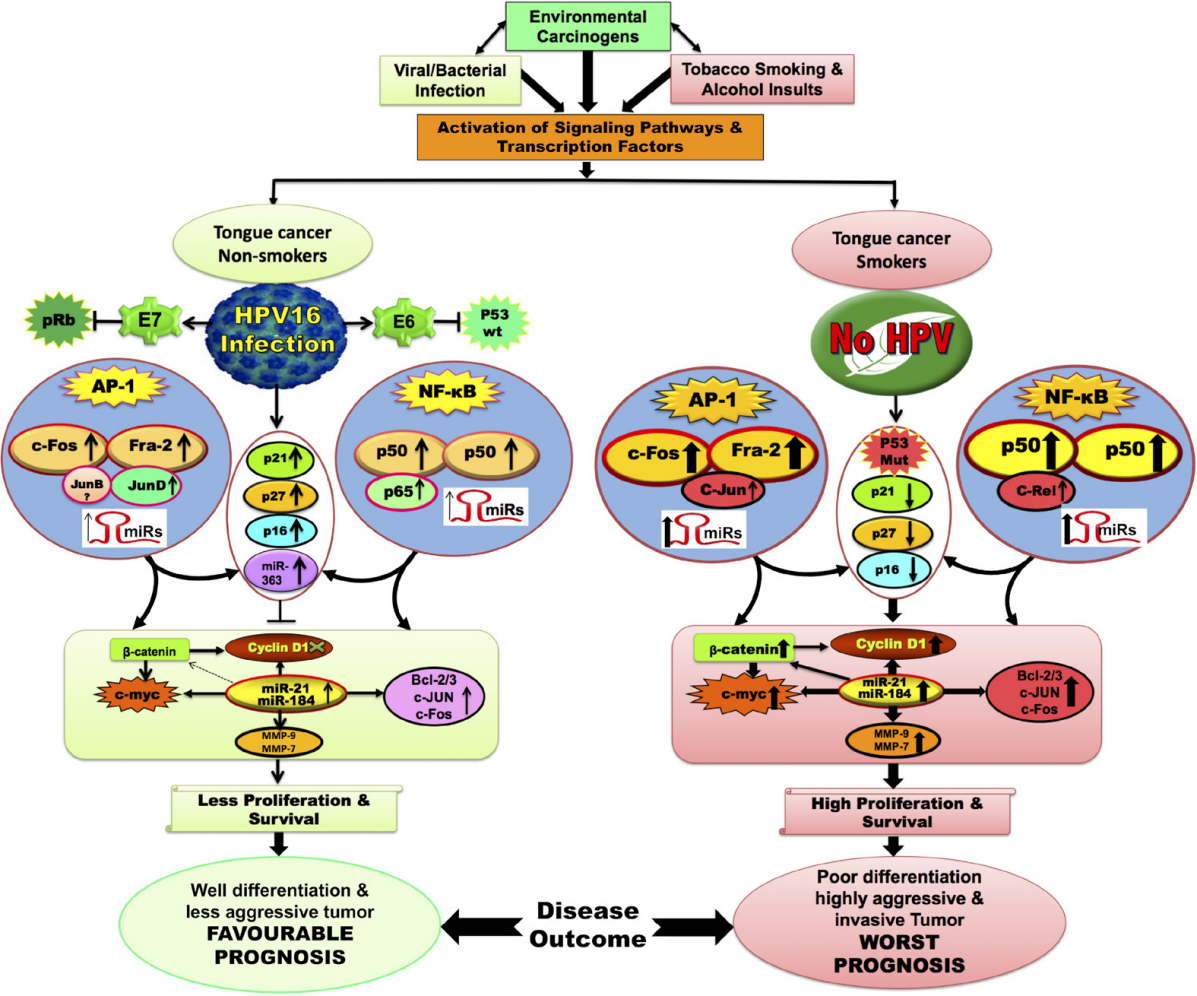
Schematic model showing the possible mechanism and cross-talk between NF-κB and AP-1 with or without HPV infection during tongue carcinogenesis Constitutive activation of AP-1; c-Fos/Fra-2 and NF-κB; p50/c-Rel play a major role during aggressive tongue carcinogenesis. Selective activation of AP-1/c-Jun and NF-κB/c-Rel in HPV negative/advanced grades of TSCCs increases the expression of its target genes (MMP-9, Bcl-2, c-myc, CyclinD1, miR-21 & miR-184) that may leads to poor differentiation and worst prognosis. Selective activation of AP-1/JunD and NF-κB/p65 in DNA-binding activity in HPV16 positive TSCC presumably increased the expression of p21, p16 & p27 which might also be contributing to better differentiation in HPV16 positive TSCCs, who show good prognosis and better survival following treatment.

All these observations put together, a model (see Figure 8) has been proposed to indicate how NF-κB, AP-1 and other associated genes involved during development, progression and prognosis of oral tongue cancer involving signalling pathways and interactions following exposure to potential risk factors; environment, tobacco, alcohol, viral (HPV) and/or bacterial infections. It suggests an essential functional regulatory role of AP-1 and NF-κB in tongue carcinogenesis both in presence or absence of HPV infection (Figure 8). Aberrant activation/expression of AP-1/Fra-2 and NF-κB/c-Rel was observed in most aggressive poorly differentiated tongue cancer with worst prognosis and the specific siRNA based silencing of c-Rel or Fra-2 further strengthened the functional role of c-Rel and Fra-2 and their possible cross-talk and interaction with other critical genes during aggressive tongue carcinogenesis.

A functional NF-κB binding site has been reported in upstream regulatory region (URR) of HPV16 that controls HPV oncogene transcription [52]. c-Rel mediated control of HPV transcription could be due to its target gene c-Jun which forms a part of functionally active AP-1, essential for HPV transcription. In turn, HPV E7 has been shown to attenuate NF-κB activation together with IκB kinase complex (Iκκ) [53]. This could be a potential reason for finding a comparatively reduced NF-κB activity in HPV^+ve^ TSCCs [22].

Earlier, we have demonstrated selective participation of c-Jun with Fra-2 led to poor differentiation and aggressive tumor phenotype mainly in HPV^−ve^ TSCCs [6]. We therefore wanted to further understand if there is any cross-talk between Fra-2 and c-Rel and for this we used Fra-2 specific siRNA and the results showed remarkable reduction in the expression of c-Rel in HPV^+/−ve^ TSCC cells (Figure 5C, 5D), while c-Rel silencing inturn led to reduced expression of Fra-2 in both HPV^+/−ve^ cells however, it was more pronounced in HPV^+ve^ cells (Figure 5A, 5B). This is because Fra-2 contains transcription binding sites for c-Rel on its promoter region and we speculate that Fra-2 might participate in modulating the expression of c-Rel through JNK/C-JUN pathway. c-Rel silencing reduced cell proliferation, invasion, and migration of TSCC cells and also inhibited expression of c-Jun, Fra-2 and c-myc.

In conclusion, it is suggested that both Fra-2 and c-Rel play essentially a critical role in the progression of TSCC specifically in HPV^−ve^ TSCC patients while HPV infection induces increased expression of p65 and p27 leading to well differentiation and better prognosis mainly in non-smoking TSCC patients.

## MATERIALS AND METHODS

### Study subjects and collection of biological samples

A total of 100 fresh tongue tissue biopsy specimens of different histopathological grades comprising precancer (*n* = 20), cancer (*n* = 50) along with adjacent normal controls (*n* = 30) were collected from the Department of ENT Surgery of Dr. Ram Manohar Lohia Hospital, New Delhi. None of these patients received any pre-operative radiation and chemotherapy. The clinico-pathological and epidemiological details of the patients were collected using standard proforma. Biopsy samples obtained immediately after surgery in sterile vials containing cold phosphate buffer saline (PBS), transported to lab on ice and stored in −80°C deep freezer till further analysis.

Written informed consent was obtained from all the subjects prior to their inclusion in the study which was carried out in accordance with the principles of the Helsinki Declaration. The study was approved by the Institutional Ethics Committee of Dr. B. R. Ambedker, Center for Biomedical Research (ACBR), University of Delhi, Delhi, India, where the study was initially carried out.

### Cell lines and cell culture

The two cell lines of tongue squamous cell carcinoma (HPV^+ve^ and HPV^−ve^) were used in the present study. HPV16^+ve^ cell line, UPCI:SCC090 was derived from the base of tongue squamous cell carcinoma [54], a kind gift from Dr. Susanne M. Gollin, University of Pittsburgh, Pittsburgh, USA. The HPV^−ve^ cell line, AW13516, a kind gift from Dr. M. M. Vaidya, ACTREC, Tata Memorial Hospital, Navi Mumbai, India was derived from squamous cell carcinoma of the human tongue [55]. Both cell lines were maintained in MEM and IMDM medium respectively, supplemented with 10% FBS and antibiotics at 37°C and 5% CO_2_.

### Electrophoretic mobility shift assay (EMSA)

EMSA was performed as described previously [6, 12]. Briefly, 10 μg of nuclear extract was incubated with γ-^32^P-radiolabeled NF-κB oligonucleotide (5′-AGTTGAGGGGACTTTCCCAGGGC-3′) for 30 min in 25 μl of reaction buffer. For the competition assay, 100× molar excess of unlabelled oligo (NF-κB) and non-specific oligo (Oct-1) was added. Protein–DNA complexes were resolved in 6% non-denaturing polyacrylamide gel and exposed to phosphorimager. For the supershift assay, 10 μg nuclear extracts in reaction buffer were incubated with specific NF-κB family protein antibodies (p50, p65, p52, c-Rel and RelB) for 60min at RT before the addition of radiolabeled probe (γ-P^32^) and electrophoresis.

### Immunoblotting

Protein isolated from all tumor tissues and cell lines (40 μg/lane) were separated on 8–15% SDS-PAGE. Immunoblotting was performed by the method described previously using specific antibodies (Supplementary Table 3) [6, 12]. The NF-κB proteins expression level was quantitated on an arbitrary scale with respect to β-actin expression as strong (+++) =>50%; Medium (++) = 10–50%; Weak (+) = < 10% and nil/not detectable (−) = < 1 as described earlier.

### siRNA interference assay

For c-Rel-siRNA interference assay, transient transfection of commercially available c-Rel and Fra-2 siRNA oligonucleotides (Santa Cruz, biotech, USA) was performed according to the manufacturer's protocol with minor modifications as described earlier [6].

### Matrigel invasion assay

Invasion assay was performed to see the effect of c-Rel silencing on invasive characteristics of TSCC cells [6]. After 24 hours of c-Rel siRNA or scramble siRNA transfection at 120 nM of concentration, TSCC cells were harvested and resuspended in FBS-free medium. A total of 1 × 10^5^ cells were plated in upper chamber of the matrigel-coated transwell (Corning, USA). Medium with serum was added to the lower chamber as a chemoattractant. After incubation for 24 hours, cells on the lower surface of the membrane were fixed with formaldehyde followed by Giemsa staining. The images of migrated cells were acquired by an inverted microscope. The number of invaded cells was quantified by counting cells in five randomly selected fields under microscope. Comparisons were made between the untreated and/or scramble-siRNA treated and c-Rel knockdown wells. All experiments were performed in triplicates.

### Migration assay

To investigate the migratory capacity of TSCC cells, cell scratch assay was performed. TSCC cells were transfected with 120 nM c-Rel siRNA and control siRNA as described earlier [6] and incubated under standard conditions to achieve knockdown of c-Rel. After 24 hours, a scratch was carefully made by scraping through each well using a sterile pipette tip (200 ul). The cells were then washed, and scratches were monitored with an inverted microscope immediately after wounding and incubation at different time points. Images were taken exactly at the same position before and after the incubation and migration rate was calculated by using image J software.

### String analysis

Protein-protein interactions (PPIs) were predicted using the STRING database v 9.1 (http://www.string-db.org/). Multiple cancer causing proteins were looked based on the following six criteria; neighbourhood, gene fusion, co-occurrence, co-expression, experimental evidence and existing databases [56].

### Statistical analysis

The data analysis was performed using the statistical software Graph Pad Prism (version 6.0). The association between HPV infection and expression profile among different histopathological grades and clinico-pathological parameters in TSCC cases was determined using Fischer's exact test and student's *t*-test (two-tailed). The *p* value ≤ 0.05 was considered as statistically significant.

## SUPPLEMENTARY MATERIALS FIGURES AND TABLES


