# Supplementary material for: Constitutive activation and overexpression of NF-κB/c-Rel in conjunction with p50 contribute to aggressive tongue tumorigenesis
Source: Oncotarget. 2018 Aug 31;9(68):33011–29. doi: 10.18632/oncotarget.26041 (PMC6152474; doi:10.18632/oncotarget.26041)
Supplement: Supplementary file 1 [file oncotarget-09-33011-s001.pdf]

Constitutive activation and overexpression of NF-κB/c-Rel in conjunction with p50 contribute to aggressive tongue tumorigenesis

SUPPLEMENTARY MATERIALS

MATERIALS AND METHODS

DNA extraction and HPV genotyping

High molecular-weight genomic DNA was isolated from precancer, cancer and adjacent normal tissues as control and tongue cancer cell lines by standard procedure of proteinase K digestion, phenol-chloroform extraction and ethanol precipitation. The initial HPV diagnosis was performed as described earlier [1]. β-globin gene was used as an internal control. HPV positive samples were subjected to comprehensive HPV genotyping assay using type-specific PCR. Primer sets are listed in Supplementary Table 1. PGMY-reverse line blot assay (RLB) was done as per recommended protocol provided by the WHO HPV LabNet program [2].

Isolation of total, cytoplasmic and nuclear proteins

Nuclear and cellular protein for EMSA and immunoblotting was isolated from tongue tissue biopsies and cell lines by the method described previously by Dignam [3] with some modifications as described earlier [4]. Briefly, the method involved fine mincing of either fresh or frozen biopsies stored at −80° C, in cold 1× PBS with the help of surgical blade in a sterile petri-dish on ice. The minced tissue material was later centrifuged at 4,000 rpm at 4° C to wash off 1× PBS solution. For preparation of total proteins, the pellet from minced tissue or different cell lines (2 × 10^6 cells) was re-suspended at 4º C for 1hour in lysis buffer [20 mM Tris (pH 7.4), 250 mM NaCl, 2 mM EDTA (pH 8.0), 0.1% Triton X-100, 0.01 mg/ml aprotinin, 0.005 mg/ml leupeptin, 0.4 mM PMSF, and 4 mM Na3VO4] followed by centrifugation at 14,000 rpm at 4º C. Alternatively, for nuclear protein, Cells (1 × 10^6) were re-suspended in ice-cold buffer A (20 mM HEPES (pH 7.6), 20% (w/v) Glycerol, 10 mM NaCl, 1.5 mM MgCl2, 0.2 mM EDTA, 1 mM DTT, 1 mM PMSF, 2 mg/ml Leupeptin and 10 mg/ml Aprotinin) and incubated on ice for 10 min with frequent vortexing. Lysate was centrifuged at 4,000 rpm for 10 min at 4º C to obtain supernatant that consisted of cytoplasmic protein. The remaining pellet containing isolated nuclei was resuspended in buffer B (20 mM HEPES (pH 7.6), 25% (w/v) Glycerol, 500 mM NaCl, 1.5 mM MgCl2, 0.2 mM EDTA, 1 mM DTT, 1 mM PMSF, 2 mg/ml Leupeptin and 10 mg/ml Aprotinin) and centrifuged after incubation for 1 h with repeated vortexing on ice at 14,000 rpm for 20 min at 4º C to obtain supernatant containing nuclear protein. The concentration of proteins isolated was determined by spectrophotometric method using Bradford dye (Bangalore Genei, Bangalore) as per protocol provided by the manufacturer and were stored in aliquots at −80º C till further use.

Total RNA extraction and RT-PCR

Total RNA was isolated from fresh tongue biopsies including pre-cancer, cancer and normal adjacent control tissues and tongue cancer cell lines using TRI reagent as per manufacturer's protocol (Sigma-Aldrich Inc, USA) [5]. For reverse transcription-PCR (RT-PCR) analysis, the isolated RNA (2 µg) was subjected to prepare cDNA using first strand cDNA synthesis kit (Fermentas, USA). RT-PCR reactions were carried out in duplicates using NF-κB subunits specific primers. The primers used for RT-PCR are listed in Supplementary Table 2. The mRNA expression levels of NF-κB genes were quantitated and normalized with respect to GAPDH which was used as an internal loading control. The PCR products were analyzed on 2% agarose gel electrophoresis.

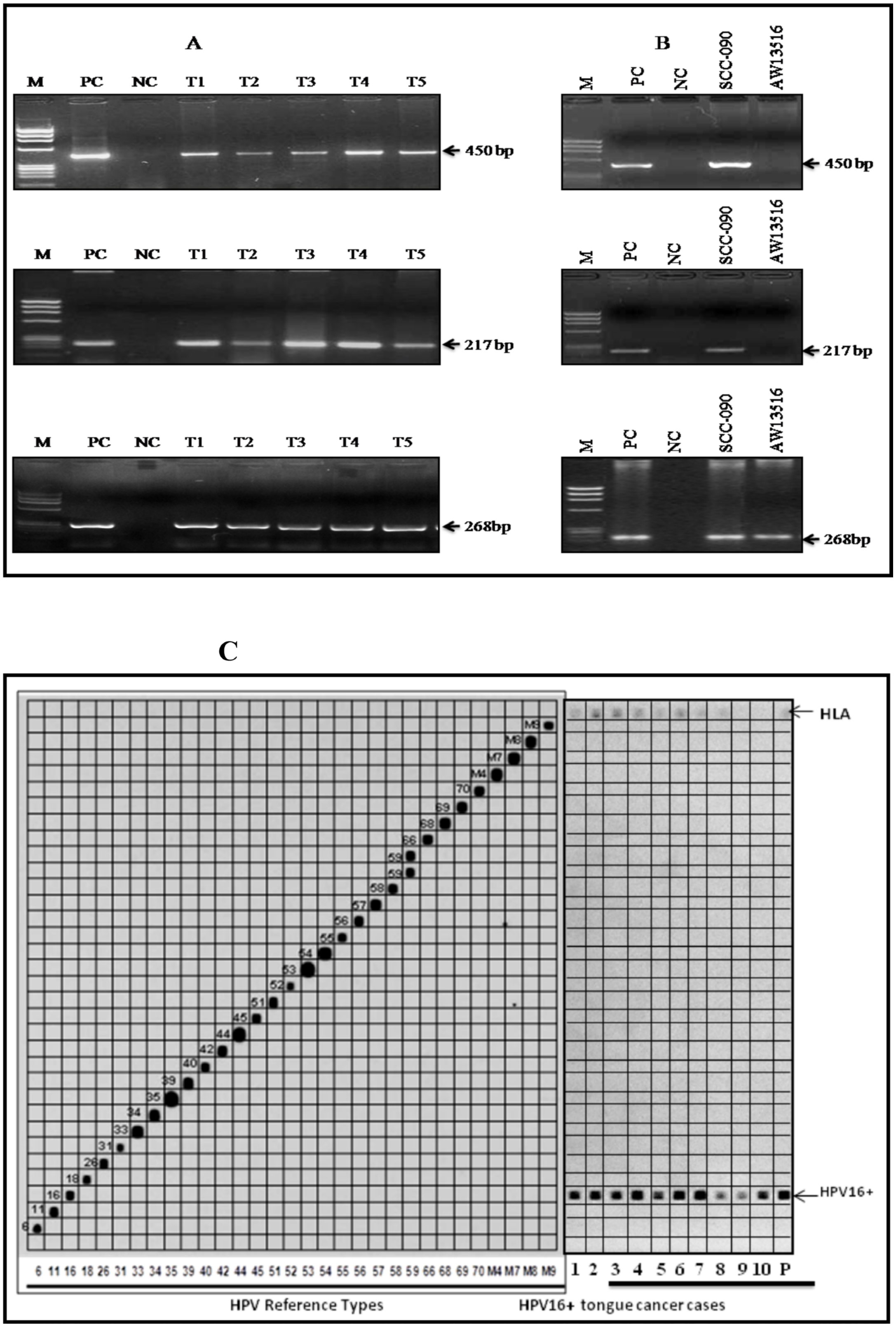
A
M PC NC T1 T2 T3 T4 T5
450 bp
217 bp
268bp
B
M PC NC SCC-090 AW13516
450 bp
217 bp
268bp
C
6 11 16 18 26 31 33 34 35 39 40 42 44 45 51 52 53 54 55 56 57 58 59 66 68 69 70 M4 M7 M8 M9
HLA
HPV16+
1 2 3 4 5 6 7 8 9 10 P
HPV Reference Types
HPV16+ tongue cancer cases

D

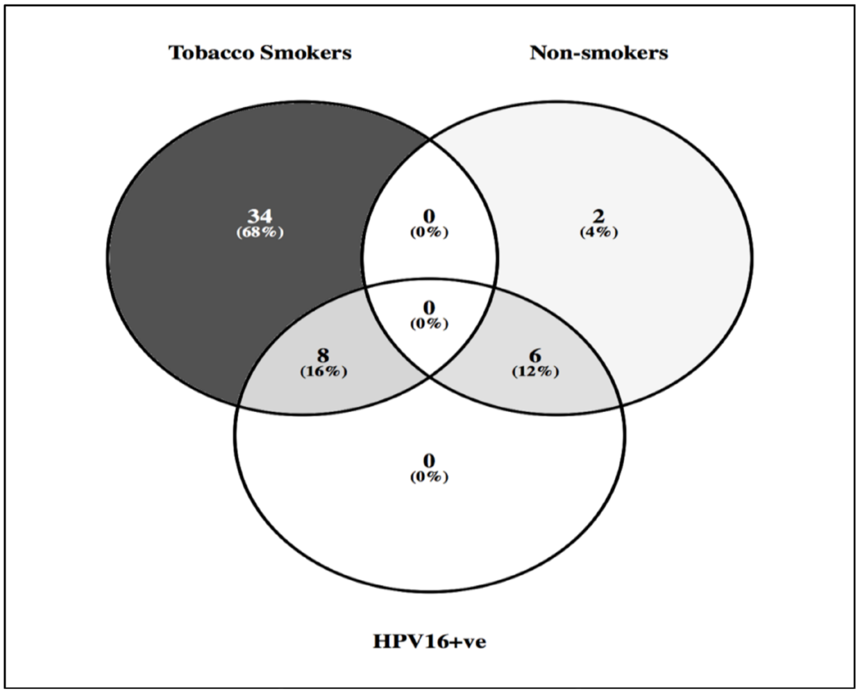

Supplementary Figure 1: (**A–D**) Detection of HPV infection & genotypes by PCR & RLB assay in TSCC cases. A and B, Representative ethidium bromide-stained 2% agarose gel showing presence of HPV infection and genotypes in tongue cancer cases (A) and cell lines (B) as indicated by appearance of L1 consensus primer-generated amplimer of 450 bp (upper panel) along with type-specific amplification of HR-HPV type 16 (217 bp) in HPV L1 positive TSCCs and cell lines (middle panel). Amplification of 268 bp fragment of β-globin gene used as internal control (lower panel). PC &, NC; are positive & negative controls respectively, Lanes T1 to 5 are tongue cancer cases, Lanes AW13516 (HPV^{-ve}) and UPCI:SCC090 ($HPV16^{+ve}$) are tongue cancer cell lines, M = φX174 HaeIII-digested molecular weight marker. (C) Representative RLB assay showing specificity of the assay for genotype identification of different HR and LR-HPV types; HPV reference types showing presence of indicated HPV types in particular lane (left panel). PGMY-RLB showing presence of exclusively the HPV16 type TSCCs (Right panel). Lane 1 to 10; HPV16 positive TSCC cases, lane PC = positive control (UPCI:SCC090 genomic DNA). (D) Venn diagram illustrating the number of HPV^{+ve} cases in the two groups; Tobacco smokers TSCCs and non-smoker TSCCs and how they overlap with each other.

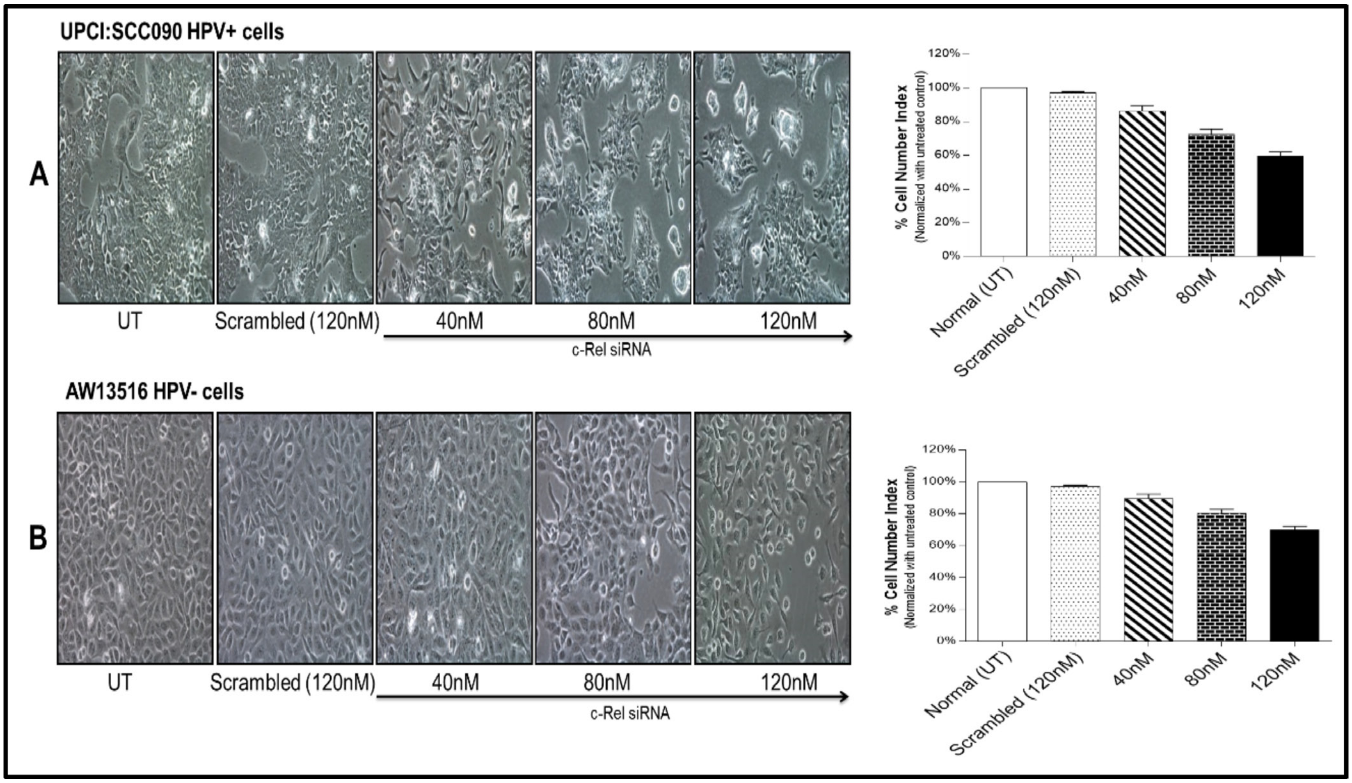

Supplementary Figure 2: (**A**–**B**) c-Rel silencing inhibits cell proliferation. c-Rel silencing was accompanied by reduction in cell viability (≥50%) at 120 nM in both (A) HPV^{+ve} (UPCI:SCC090) and (B) HPV^{-ve} (AW13516) TSCC cells. Semi-confluence cancer cells transfected with indicated concentrations of c-Rel siRNA and scrambled control for 48 hrs and were harvested by trypsinization and counted for live cells using trypan blue dye. Panels a & b indicate photomicrographs of respective controls and treated cells in culture. Data represent the means ± S.D. of triplicate cultures.

Tables 1 and 2: Primer sequences used for detection and typing of HPV sequences and mRNA transcripts analysis of AP-1 family genes

Table 1

Primers	Primer sequence
**MY09/MY11**	FP- 5′ CGTCCMARRGGAWACTGATC-3′<br>RP- 5′ GCMCAGGGWCATAAYAATGC-3′<br>Where M = A or C, W = A or T, Y = C or T and R = A or G
**HPV16(URR)**	FP- 5′ AAGGCCAACTAAATGTCAC 3′<br>RP- 5′ CTGCTTTTATACTAACCGG 3′
**β-globin**	FP- 5′ GAAGAGCCAAGGACAGGTAC-3′<br>RP- 5′ CAACTTCATCCACGTTACACC-3′

Table 2

RT-Primers	Primer sequence
**p50**	FP- 5′CACTTATGGACAACTATGAGGTCTCTGG 3′<br>RP- 5′CTGTCTTGTGGACAACGCAGTGGAATTTTAGG 3′
**P65**	FP- 5′AGCACAGATACCACCAAGACCC 3′<br>RP- 5′CCAGGGAGATGCGCACTG 3′
**c-Rel**	FP- 5′-TTACCAGAAATGCCCAGGTC -3′<br>RP- 5′-AGGCCCTTCTAGGAATGGAA-3′
**GAPDH**	FP- 5′ TGGATATTGTTGCCATCAATGACC-3′<br>RP- 5′ GATGGCATGGACTGTGGTCATG-3′

Table 3: List of antibodies used in the present study

S. No.	Antibodies	Clonality	Manufacturer	Catalogue No.
1.	p50	Rabbit polyclonal	Santa Cruz	sc-7178
2.	p52	Mouse monoclonal	Santa Cruz	sc-7386
3.	p65	Rabbit polyclonal	Santa Cruz	sc-109
4.	c-Rel	Rabbit polyclonal	Santa Cruz	sc-70
5.	RelB	Rabbit polyclonal	Santa Cruz	sc-226
6.	Fra-2	Rabbit polyclonal	Santa Cruz	sc-13017
7.	HPV16 E6	Mouse monoclonal	Santa Cruz	sc-460
8.	HPV16 E7	Mouse monoclonal	Santa Cruz	sc-264
9.	β Actin	Mouse monoclonal	Santa Cruz	sc-47778

REFERENCES

1. Mishra A, Bharti AC, Varghese P, Saluja D, Das BC. Differential expression and activation of NF-kappaB family proteins during oral carcinogenesis: Role of high risk human papillomavirus infection. Int J Cancer. 2006; 119:2840–50. https://doi.org/10.1002/ijc.22262.
2. WHO. Human papillomavirus laboratory manual: World Health Organization, Geneva. 2010.
3. Dignam JD. Preparation of extracts from higher eukaryotes. Methods Enzymol. 1990; 182:194–203. https://doi.org/0076-6879(90)82017-V.
4. Rosl F, Das BC, Lengert M, Geletneky K, Zur Hausen H. Antioxidant-induced changes of the AP-1 transcription complex are paralleled by a selective suppression of human papillomavirus transcription. J Virol. 1997; 71:362–70.
5. Gupta S, Kumar P, Kaur H, Sharma N, Saluja D, Bharti AC, Das BC. Selective participation of c-Jun with Fra-2/c-Fos promotes aggressive tumor phenotypes and poor prognosis in tongue cancer. Scientific Reports. 2015; 5:16811.
